# 

**Published:** 1961-10

**Authors:** 


### Contents

THE UNIVERSITY AND THE MEDICAL SCHOOL *Sir Philip Morris* 103

FUNGUS INFECTIONS OF THE SKIN IN BRISTOL AND THE WEST COUNTRY . . . . . . . *Mary P. English* 112

THE PROBLEM OF HEADACHE . . . . . *F. T. Page* 122

BRONCHOPNEUMONIA DUE TO *PASTEURELLA MULTOCIDA* (*SEPTICA*) . . . . . . *G. A. Hayes and G. Neale* 129

ANKYLOSING SPONDYLITIS WITH MITRAL STENOSIS *Clinico—Pathological Conference* 132

NOTES AND NEWS . . . . . . . . 143

APPOINTMENTS . . . . . . . . 144

NEWS FROM THE SOCIETIES . . . . . . . 146

